# Host gill attachment causes blood-feeding by the salmon louse (*Lepeophtheirus salmonis*) chalimus larvae and alters parasite development and transcriptome

**DOI:** 10.1186/s13071-020-04096-0

**Published:** 2020-05-06

**Authors:** Erna Irene Heggland, Michael Dondrup, Frank Nilsen, Christiane Eichner

**Affiliations:** 1 Department of Biological Sciences and Sea Lice Research Centre (SLRC), University of Bergen, Bergen, Norway; 2 Department of Informatics and Sea Lice Research Centre (SLRC), University of Bergen, Bergen, Norway

**Keywords:** Salmon louse, Ectoparasite, Blood-feeding, Hematophagy, Gills, RNA-sequencing

## Abstract

**Background:**

Blood-feeding is a common strategy among parasitizing arthropods, including the ectoparasitic salmon louse (*Lepeophtheirus salmonis*), feeding off its salmon host’s skin and blood. Blood is rich in nutrients, among these iron and heme. These are essential molecules for the louse, yet their oxidative properties render them toxic to cells if not handled appropriately. Blood-feeding might therefore alter parasite gene expression.

**Methods:**

We infected Atlantic salmon with salmon louse copepodids and sampled the lice in two different experiments at day 10 and 18 post-infestation. Parasite development and presence of host blood in their intestines were determined. Lice of similar instar age sampled from body parts with differential access to blood, namely from gills *versus* lice from skin epidermis, were analysed for gene expression by RNA-sequencing in samples taken at day 10 for both experiments and at day 18 for one of the experiments.

**Results:**

We found that lice started feeding on blood when becoming mobile preadults if sitting on the fish body; however, they may initiate blood-feeding at the chalimus I stage if attached to gills. Lice attached to gills develop at a slower rate. By differential expression analysis, we found 355 transcripts elevated in lice sampled from gills and 202 transcripts elevated in lice sampled from skin consistent in all samplings. Genes annotated with “peptidase activity” were among the ones elevated in lice sampled from gills, while in the other group genes annotated with “phosphorylation” and “phosphatase” were pervasive. Transcripts elevated in lice sampled from gills were often genes relatively highly expressed in the louse intestine compared with other tissues, while this was not the case for transcripts elevated in lice sampled from skin. In both groups, more than half of the transcripts were from genes more highly expressed after attachment.

**Conclusions:**

Gill settlement results in an alteration in gene expression and a premature onset of blood-feeding likely causes the parasite to develop at a slower pace.
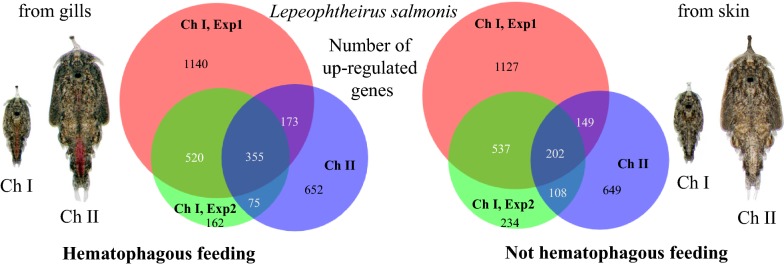

## Background

The salmon louse, *Lepeophtheirus salmonis* (Krøyer, 1837) (Crustacea: Caligidae) and its Atlantic subspecies *L. salmonis* [1], is an obligate ectoparasite of salmonid fish, such as the Atlantic salmon (*Salmo salar*). The parasite is of major concern for the aquaculture sector in the Northern Hemisphere, as it causes challenges for the industry with its high fecundity and resistance towards several chemotherapeutants [2]. The parasite life-cycle consists of both planktonic and parasitic stages [3, 4]. Upon hatching from a fertilized egg, the parasite is in the nauplius I stage. Thereafter, the salmon louse molts into the nauplius II stage, and further to the infective copepodid stage. Successive molting occurs on the host, first to the parasitic chalimus I and II. These stages are attached to the host by their elongated frontal filament [5, 6], and are therefore immobile. Another molting renders the parasite mobile, as it is no longer secured by the frontal filament, but holds itself by using its cephalothorax as a suction cup. These stages are the preadult I and II and adult lice. However, during the molt to next stage, also mobile lice attaches to the host by a frontal filament. The mobile parasite grazes on larger parts of its host, selecting its preferred feeding site and causes greater damage to the fish [7, 8]. Progression of the salmon louse life-cycle is temperature dependent, and at 10 °C, the time from fertilization to mature adult is approximately 40 (male) to 52 (female) days [9], or 38 (male) to 44 (female) days for the fastest developers [10].

The alimentary canal of the salmon louse develops during the copepodid stage [11]. The alimentary canal is composed of a mouthpart, an esophagus, a midgut, and a hindgut ending in a short rectum [11, 12]. Vertebrate blood is a highly nutritious tissue fluid that is constantly renewed. Hematophagy (blood-feeding behavior) is therefore a common strategy among parasitizing arthropods. The diet of the salmon louse consists of the skin and blood of its host [13], and the blood-filled intestine is visible as a red line throughout the its body. Upon ingestion of blood, hematophagous parasites need to express genes encoding proteins that can manage the blood components. Blood is particularly enriched in proteins that contain the pro-oxidant molecules heme and iron. These are essential cofactors for the salmon louse, yet also highly toxic if not bound and detoxified by chaperones. Therefore, the alimentary canal needs to withstand, digest and absorb components of the food bolus. Trypsin-like enzymes [14, 15], a lipid transfer protein [16], a putative heme scavenger receptor [17] and the iron storage units of ferritin [18] are all expressed in the salmon louse midgut.

The distribution of copepodids on wild and farmed hosts shows that the preferred settlement sites are on the fins and the scaled body of the host [5]. Some groups have reported the settlement of lice on gills as well; however, this is considered rather uncommon (reviewed by Treasurer et al. [19]). In laboratory trials, on the other hand, lice are often found on gills, although there still seems to be a higher preference for the fins and body [7, 19]. Copepodid gill settlement is therefore often considered an experimental artefact due to altered host behavior during laboratory infestations [19]. Gill tissue in teleost fish is highly vascular, whereas skin epidermis is not. The chalimus frontal filament, appendages and mouth tube have been shown to not breach the basement membrane within the salmon skin [20], thus do not reach the dermal vascular layer. Salmon lice settling on gills might therefore be more prone to ingest a blood meal than those lice elsewhere on the host during early stages of attachment.

The genome of the Atlantic salmon louse is fully sequenced and high-throughput transcriptome studies have been conducted under various experimental conditions using microarrays as well as sequencing. Examples of such experimental settings include host-parasite interactions on different hosts [21], hosts fed different diets [22], response to drugs [23], larval stress response [24], parasite sex differences [25] and development [26]. Recently, we have used RNA-sequencing (RNA-seq) to investigate patterns of gene expression during molting in the parasitic larval stages of *L. salmonis* [27]. Transcriptome plasticity in response to hematophagy has been investigated in various arthropods for which controlled blood-feeding is possible. Arthropod species subjected to such controlled feeding trials include mosquitoes (*Aedes* spp. [28–30] and *Anopheles gambiae* [31]), the biting midge *Culicoides sonorensis* [32] and ticks (*Ixodes* spp. [33, 34]). However, investigating transcriptional changes induced by a blood meal within the salmon louse is challenging, as no protocol for feeding lice *in vitro* exists. To overcome this limitation, equally developed lice of the same batch, infecting the same fish, were sampled from host body attachment sites with predicted differing access to blood.

In this study, we infected Atlantic salmon with salmon louse copepodids and sampled the lice on the 10th and 18th day post-infestation (dpi), when the lice were in the chalimus I and chalimus II stage or had recently molted to the preadult I stage. Parasite settlement site and visible presence of host blood in louse intestines were recorded. Transcriptomes of equally developed lice sampled from different locations (gills and the body/fins), representing lice with access to blood *versus* lice without access at 10 and 18 dpi, were examined by RNA-sequencing. Specific aims of this study were to investigate: (i) visible blood ingestion from various sampling locations; (ii) development of lice from locations differing in blood access; and (iii) differences in gene expression of immobile lice from locations with unequal access to blood.

## Methods

### Animals

Atlantic salmon lice (*L. salmonis salmonis*) [1] were raised on Atlantic salmon in flow through tanks with seawater (salinity 34.5‰ and temperature 10 °C) [35]. A laboratory strain of *L. salmonis* called LsGulen [35] was used. Fish were handfed commercial dry pellets daily and maintained according to Norwegian animal welfare regulations. Fish were anesthetized by a mixture of methomidate (5 mg/l) and benzocaine (60 mg/l) prior to handling. For sampling of early developmental stages of lice, fish were killed by a swift blow to the head. Salmon louse egg string pairs were incubated and hatched in incubators in a seawater flow through system [35]. Emerging copepodids were used to infect fish in 500-liter tanks. Copepodids between 4–14 days post-hatching were used. Fish were infected with approximately 70 copepodids per fish. The number of copepodids used was estimated as described by Hamre et al. [35]. Prior to infestation, the tank water was lowered and copepodids spread on the surface.

### Sampling of lice

At 10 and 18 dpi, fish were sacrificed, and lice were removed with forceps and photographed for subsequent measurements. The gills were cut out and observed under a microscope. Any lice present were sampled, photographed and placed in RNAlater in individual tubes. At 10 dpi 20 and 18 fish in Experiment 1 and 2 were sampled, respectively, at 18 dpi 37 and 34 fish for Experiment 1 and 2, respectively. Measurements of all lice were done on photographs. Total length (TL) and cephalothorax length (CL) were measured as earlier described [27, 36] enabling the determination of the developmental status as well as sex differentiation for the chalimus II larvae using TL and CL measurements as described previously [27, 36]. In short, the total length of lice and the length of the cephalothorax were measured on the photographs. As the ratio between these two measurements decreases with instar age, it can be used as an approximation of the instar age. Lice, which had recently molted into a stage are called young, in the middle of a stage middle and lice soon molting to the next stage are called old. In addition, measurable sex differences of the cephalothorax length of chalimus II lice can be used to distinguish between the sexes at this stage. Preadult lice sampled at 18 dpi from 10 (Experiment 1) or 9 fish (Experiment 2), were investigated for the existence of a frontal filament using the photographs. For RNA isolation prior to RNA-seq, lice were sorted into groups of equal developmental status as well as sex in case of chalimus II (were sex discrimination is possible) as described by Eichner et al. [27]. We used lice which were equal both within each group and between groups from different sampling locations (gills, skin). All lice analyzed were lice of old instar age (shortly before molting). All chalimus II lice were female. Five chalimus I or four chalimus II lice, respectively, were pooled together into one sample. For RNA-seqencing, RNA from both experiments sampled at 10 dpi and from one experiment sampled at 18 dpi was analyzed. Eight (Experiment 1, 10 dpi), six (Experiment 2, 10 dpi) or five (Experiment 2, 18 dpi) replicates were analyzed per group (lice sampled from gills, lice sampled from skin), giving rise to 38 samples in total.

### RNA isolation and sequencing

RNA was isolated as described before [37]. In brief, pools of four or five chalimus larvae were homogenized in TRI reagent and mixed with chloroform (both Merck, Darmstadt, Germany). The upper aqueous phase was aspirated and further purified using an RNeasy Micro Kit (Qiagen, Hilden, Germany) for RNA isolation according to the manufacturers’ instructions. RNA was stored at − 80 °C until use. Library preparation and RNA-sequencing were conducted by the Norwegian Sequencing Centre, Oslo, as previously described [27]. Briefly, sequencing libraries were prepared from 0.5 µg total RNA using the TruSeq stranded mRNA reagents (Illumina, San Diego, USA). Indexed libraries were blended into a single pool and sequenced during three runs of a NextSeq 500 instrument (Illumina) using 76-bp single end reads. Image analysis and base calling were performed using Illumina’s RTA software version 2.4.11, and data were converted to FASTQ format using bcl2fastq version 2.17.1.14. Raw sequencing data were deposited in the NCBI database under BioProject ID PRJNA577842.

### Data processing of RNA-sequencing data

Obtained sequences were quality controlled by FastQC v.0.11.5 [38]. Reports were summarized using MultiQC 1.0 [39]. For reference sequences, we used a combination of the Ensembl Metazoa reference assembly [40] of the nuclear genome (*Lepeophtheirus salmonis*, LSalAtl2s) and the mitochondrial genome RefSeq sequence NC_007215.1 [41]. The gene models from Ensembl Metazoa were further augmented with gene models derived from full-length sequences of LsFer1 and LsFer4 obtained by rapid amplification of cDNA ends (RACE) [18], by aligning the RACE consensus sequences against the nuclear assembly with GMAP [42]. RNA-seq reads were aligned against the reference using the STAR aligner [43]. Then, alignments were sorted and indexed using SAM-tools [44] and saved in BAM format. Technical replicates were merged prior to counting using the merge function in SAM-tools. RNA-seq reads and their overlap with annotated nuclear and mitochondrial transcripts were counted using the software featureCounts [45] with settings for strand-specific reverse stranded libraries.

Differential expression (DE) analysis was done with DESeq2 [46] on raw counts using Galaxy [47] under the Norwegian e-Infrastructure for Life Sciences (NeLS) platform [48]. A false discovery rate (FDR) adjusted *P*-value (*P*adj) of less than 0.05 was considered as significant. Prior to DE analysis, all transcripts with less than four counts in all samples were removed. The same dataset was also analyzed with EdgeR [49] (Additional file 1: Table S1). Also here a significance cut-off of FDR adjusted *P*-value of less than 0.05 was used. Because the results from the two methods are in near perfect agreement, we further refer to the results produced by DESeq2. Venn diagrams were prepared using the BioVenn platform (http://www.biovenn.nl/) [50]. Hierarchical clustering as well as GO annotation enrichment were performed in J-Express [51, 52]. GO terms were summarized in REVIGO [53].

### Transcript annotation

Protein-coding transcripts were annotated by running NCBI-Blast+, BlastP version 2.6.0+ [54, 55] of their corresponding predicted Ensembl protein sequences against the GenBank (NR) [56] and SwissProt [57] databases. Gene Ontology (GO) terms (full terms and GOslim annotation) and protein families (Pfam) were automatically assigned by InterProScan 5 [58].

## Results

### Distribution and characteristics of lice

Upon termination, all lice were removed from the salmon, and their settlement site and developmental stage and the visibility of an intestine filled with blood were assessed. The average number of lice on the different body parts is shown in Table 1.

**Table 1:** Average numbers of lice on different body parts of the fish sampled at 10 and 18 days post-infestation in Experiment 1 and Experiment 2

Body part	Experiment 1		Experiment 2	
	10 dpi	18 dpi	10 dpi	18 dpi
Body	8.5	15.8	9.7	26.2
Fins	11.5	4.1	13.2	6.3
Gills	6.5	4.2	10.0	10.1
Total	26.4	24.1	32.9	42.6

**Fig. 1:**
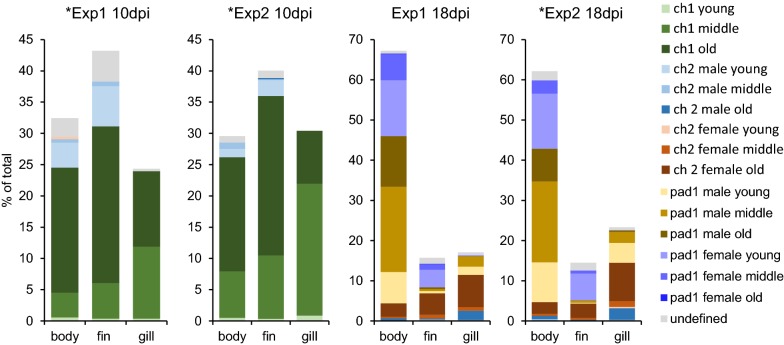
Distribution of different stages and instar ages of lice. Stages and different instar ages of lice sampled at 10 days post-infestation (dpi) and at 18 dpi sampled from the fish body, fins and gills in both experiments (Experiment (Exp) 1 and Experiment 2) are shown. Number of fish sampled: *n* = 20 (Experiment 1, 10 dpi), *n* = 18 (Experiment 2, 10 dpi), *n* = 37 (Experiment 1, 18 dpi) and *n* = 34 (Experiment 2, 18 dpi). Lice instar ages were defined on photographs as described in the main text. Transcriptome sequencing was performed for lice from Experiment 1 10 dpi, Experiment 2 10 dpi (both chalimus I old) and Experiment 2 18 dpi (chalimus II old) marked with *

Figure 1 depicts the distribution of different developmental stages and instar ages of lice on the host at 10 and 18 dpi. At 10 dpi, most lice were attached to the fins, but there was also a high percentage of lice on the body and the gills. At 18 dpi, however, the highest percentage of lice was found on the body of the salmon. Different stages were distributed differently between sites.

At 10 dpi, the highest percentage of lice was in the chalimus I stage (83% and 95% in Experiment 1 and Experiment 2, respectively) while the remaining lice were chalimus II. The chalimus II larvae (mainly males) were found on the body and fins, but none on gills. On the gills more chalimus I larvae were found (more middle stage than old) (Fig. 1, Table 1).

At 18 dpi, there were predominantly preadult I lice (75% or 74% in Experiment 1 and Experiment 2, respectively). On the body and fins, most lice were at the preadult I stage, while on the gills, most lice were in the chalimus II stage. Preadult I females were found on the body and fins, but almost none on the gills (< 1%). On the gills, on the other hand, there was a higher percentage of old chalimus II females. There was a higher proportion of preadult I male lice found on the body compared with the fins. On the fins, most of the preadult I lice were young females (Fig. 1, Table 2).

**Table 2:** Distribution of *Lepeophtheirus salmonis* stages at sampling time points

Body part	Chalimus I	Chalimus II		Preadult I		NN
		Male	Female	Male	Female	
Experiment 1 (10 dpi)						
Body	75.6	14.0	1.2	–	–	9.3
Fins	72.1	16.6	–	–	–	11.4
Gills	98.4	–	–	–	–	1.6
Experiment 2 (10 dpi)						
Body	88.6	8.0	–	–	–	3.4
Fins	89.9	7.2	–	–	–	3.0
Gills	100.0	–	–	–	–	–
Experiment 1 (18 dpi)						
Body	–	1.0	5.5	61.9	30.5	1.0
Fins	–	3.6	40.1	9.5	37.2	9.5
Gills	–	14.8	52.3	27.5	1.3	4.0
Experiment 2 (18 dpi)						
Body	–	2.0	5.5	61.5	27.3	3.7
Fins	–	1.9	27.9	5.8	51.0	13.5
Gills	–	13.5	48.8	34.4	0.3	3.0

*Notes*: The percentage of different stages and sexes (for chalimus II and preadult I) of lice sampled from the specific body parts of all fish at 10 days post-infestation (dpi) and 18 dpi in Experiment 1 and Experiment 2 are shown

*Abbreviation*: NN, undefined

**Fig. 2:**
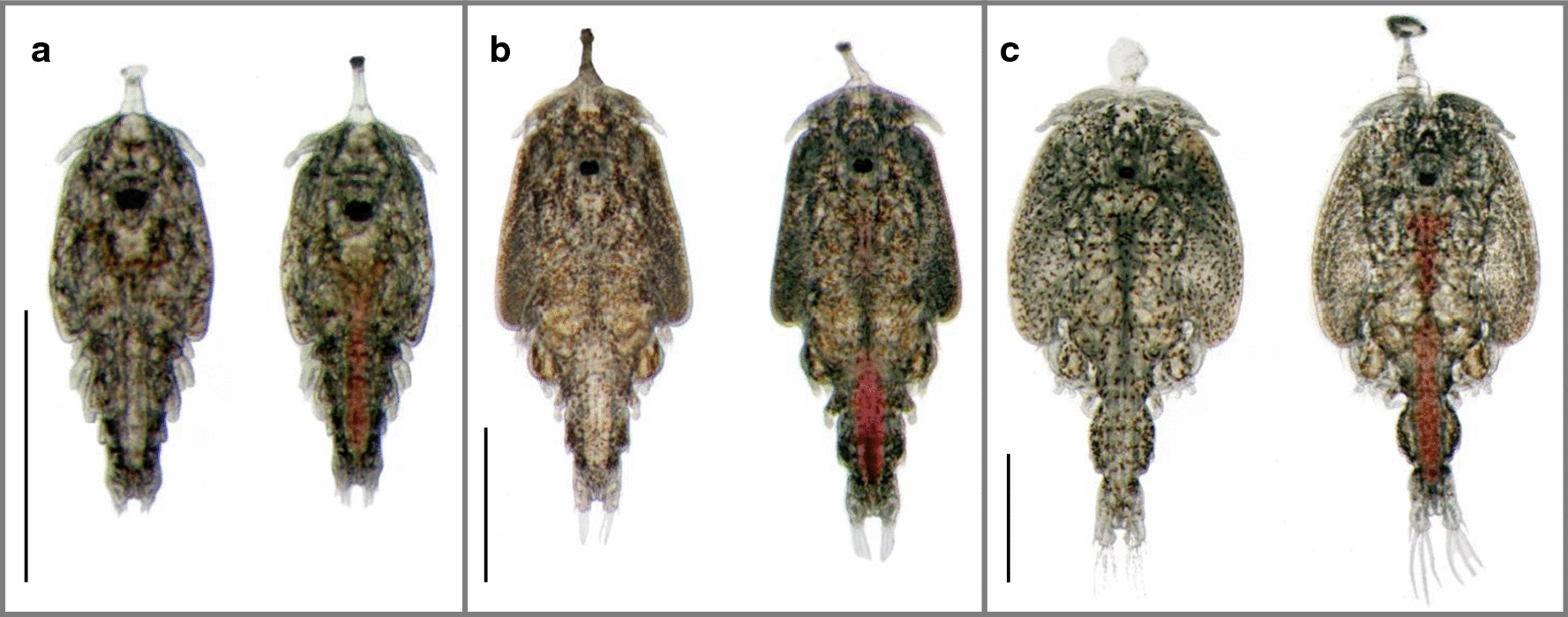
Photographs of salmon lice with (right) and without (left) a blood-filled intestine. **a** Chalimus I larvae sampled 10 days post-infestation. **b** Chalimus II larvae sampled 18 days post-infestation. **c** Preadult I lice on frontal filament sampled 18 days post-infestation. The lice with blood-filled intestine were sampled from the gills and the others were sampled from the skin of their host. *Scale-bars*: 1 mm

For preadult I lice from 10 or 9 fish in Experiment 1 and Experiment 2, respectively, the presence of a frontal filament was also investigated. Presence of a frontal filament and a visible blood-filled intestine with respect to the settlement site are summarized in Table 3. The majority of the preadult I lice were located on the host body, and the minority was located on the gills. However, the preadult I lice on the gills were more often secured by their frontal filament. Of the preadult I lice still attached by the filament, only the ones on the gills had a blood-filled intestine (Table 3, Fig. 2c). None of the lice on the fins had a blood-filled intestine, and on the host body, only the mobile lice had apparently fed on blood. Additionally, we found both chalimus I (Fig. 2a) (at 10 dpi) and chalimus II (Fig. 2b) larvae attached to the gills that had fed on blood, whereas lice of the same age on the fins and body had no visible blood in the intestine.

**Table 3:** Distribution of preadult *Lepeophtheirus salmonis* at 18 days post-infestation

Group of lice	Experiment 1				Experiment 2			
	All	Body	Fins	Gills	All	Body	Fins	Gills
Pad I of total	68 (294)	88 (228)	49 (40)	30 (26)	66 (329)	87 (206)	63 (55)	38 (62)
Female of pad I	33 (102)	30 (68)	78 (31)	8 (2)	35 (115)	30 (62)	87 (48)	0 (0)
Pad I on filament	10 (29)	5 (12)	25 (10)	27 (7)	21 (69)	6 (12)	38 (21)	58 (36)
Pad I with visible blood	16 (48)	13 (30)	0 (0)	69 (18)	24 (80)	13 (27)	0 (0)	85 (53)
Pad I on filament with visible blood in intestine	1 (2)	0 (0)	0 (0)	8 (2)	9 (30)	0 (0)	0 (0)	48 (30)

*Notes*: Preadult (pad) I lice sampled from 10 and 9 fish (Experiment 1 and 2, respectively) were investigated in more detail. The percentage and absolute numbers (in parentheses) of pad I lice sampled from all body parts of the fish (all) and separated into different sampling places (body, fins, gills) are shown for Experiment 1 and 2. The table shows the overall percentage of pad I of all lice (pad I and chalimus II), what percentage of these were females, the percentage of pad I lice found on a filament, the percentage of lice with visible blood in the intestine as well as the percentage of pad I lice with both, blood filled intestine and a filament

### Transcriptome sequencing

Illumina sequencing of mRNA produced 719 million single-end reads with a length of 76 bases and a total of 54.6 billion bases sequenced. Out of all reads, 633 million (88%) aligned uniquely to the reference and 41.6 million (5.79%) aligned to multiple genomic loci. Further, 511.1 million reads (78.35%) of the aligned reads overlapped with exon regions of the annotated gene models.

### Effect of gill settlement on the transcriptome in chalimus larvae

**Fig. 3:**
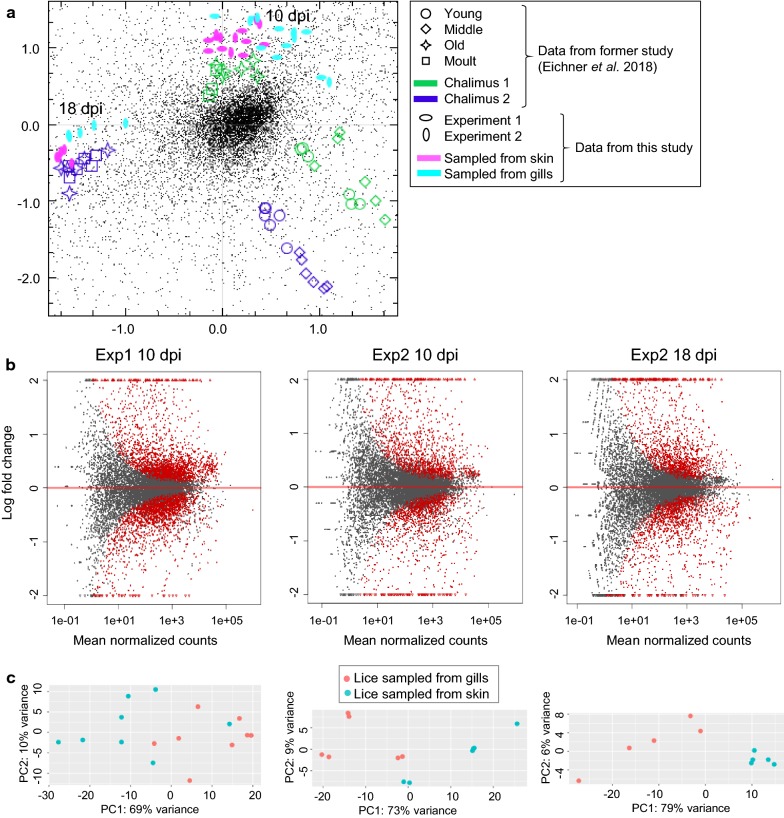
Overall differences in gene expression between different groups of lice. **a** Correspondence analysis (CA) plot showing the overall gene expression of the samples analysed in this study (pink and turquoise dots) in comparison with other chalimus I and chalimus II larvae divided into various instar ages taken from Eichner et al. [27]. **b** MA plots for DESeq2 comparing the different conditions (lice sampled from gills *versus* lice sampled from skin at 10 days post-infestation (dpi) and 18 dpi, respectively) in the samplings: Experiment (Exp) 1, sampled at 10 dpi, Experiment 2 sampled at 10 dpi and Experiment 2 sampled at 18 dpi. The average binary logarithm of the expression across all samples is shown on the x-axis and the binary logarithm of fold change is shown on the y-axis. Red dots indicate differentially expressed genes (DESeq2, *P*adj < 0.05) while grey dots are not differentially expressed between the two groups. **c** Principal components analysis (PCA) plots of the same data

In order to determine the effect of gill settlement on the gene expression in chalimus larvae, RNA-sequencing of pooled individuals of equal development was performed. All counts per million (CPM) values can be found in Additional file 2: Table S2. The overall gene expression of the individual samples in comparison with chalimus I and chalimus II larvae of different instar age (data taken from Eichner et al. [27]) is shown in a correspondence analysis (CA) plot (Fig. 3c).

All lice from this study sampled at 10 dpi clustered together with chalimus I larvae sampled directly before molting as well as molting ones (old, molt) from Eichner et al. [27] and all lice sampled at 18 dpi from this study clustered with chalimus II lice sampled directly before molting. Lice sampled from gills and lice sampled from skin differed also slightly in their overall gene expression. Lice from Experiment 1 at 10 dpi clustered together with lice from the respective group (from gills or from skin) at 10 dpi in Experiment 2. This indicates that lice sampled at 10 dpi in the different experiments were composed of batches of lice of comparable instar age. DE analyses were performed for each sampling separately. MA plots as well as a principal components analysis (PCA) plots for each sampling are shown in Fig. 3a, b. A list of all genes with log2 fold changes and false discovery rate (FDR) adjusted *P*-values (*P*adj) for each sampling can be found in Additional file 1: Table S1. A total of 5878 genes were differentially expressed in at least one of the samplings (Additional file 3: Table S3) (DESeq2: *P*adj < 0.05).

**Fig. 4:**
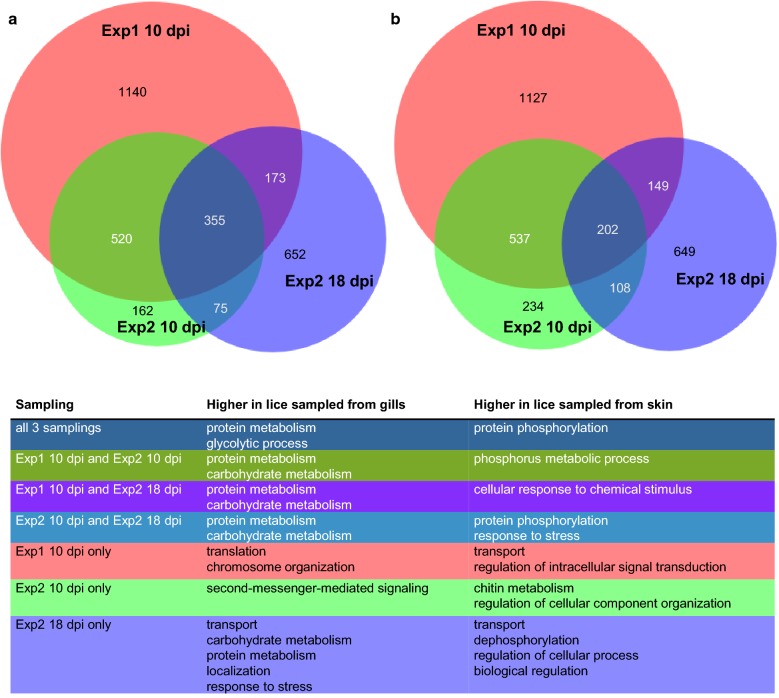
Scaled Venn diagrams for differentially expressed genes found in lice from skin and from gills. The Venn diagrams are showing the number of differentially expressed genes (DESeq2; *P*adj < 0.05) between chalimus larvae sampled from gills *versus* those sampled from skin, for Experiment (Exp) 1, sampled at 10 days post-infestation (dpi), Experiment 2, sampled at 10 dpi and Experiment 2 sampled at 18 dpi. The numbers of genes elevated in chalimus larvae sampled from gills in the different samplings are shown in (**a**) and the numbers of genes elevated in chalimus larvae sampled from fish skin in the different samplings are shown in (**b**). Representative GO terms for each group are given in the table in the bottom panel. A full list of enriched GO terms is shown in Additional file 15: Table S9 which are summarized as Tree maps in Additional file 1: Figure S1

The most DE genes were found in Experiment 1 at 10 dpi (2188 or 2015 upregulated in samples from gills or skin, respectively). In Experiment 2 at 10 dpi, only 1112 or 1081 transcripts for samples from gills or skin, respectively, of which 79% (skin) or 68% (gills), overlapped with the ones found in Experiment 1 at 10 dpi. DE genes found at 18 dpi overlapped somewhat less with DE genes found in Experiment 1 at 10 dpi. Only 43% or 32%, respectively, of the genes found here overlapped with genes from the respective groups in Experiment 1 at 10 dpi and 35% or 28%, respectively, overlapped with Experiment 2 at 10 dpi (Fig. 4). There were 616 genes, which were DE in all three samplings. Of these 355 were elevated in lice samples from gills (Additional file 4: Table S4) and 202 were elevated in lice samples from skin (Additional file 5: Table S5). The remaining 59 genes were significant (DESeq2, *P*adj < 0.05) different, but regulation directions differed between time points (31 elevated in lice from gills at 10 dpi but lower at 18 dpi, 24 the other way around and 4 differed between lice sampled at day 10 in the two different experiments) (Additional file 6: Table S6). Transcripts solely regulated at either 10 dpi or 18 dpi are listed in Additional file 7: Table S7 and Additional file 8: Table S8, respectively.

Among DE genes found at 10 dpi or at 18 dpi we found a high number of genes (51) annotated with Pfam domain PF00040: “Fibronectin type II domain”. These were mostly elevated in lice sampled from gills at 10 dpi. However, a smaller number of PF00040 is under the DE genes which were elevated in lice sampled from skin compared to gills. Additionally, 85 transcripts with Pfam domain PF00089: “Trypsin” were under the DE genes and were mostly found upregulated in the group of lice sampled from gills.

The sizes and overlaps of gene sets that were DE in each experiment separated by expression pattern (elevated in lice sampled from gills or elevated in lice sampled from skin) are depicted in Venn diagrams together with most representative GO terms in Fig. 4. All significantly enriched GO terms (*P*-value < 0.05) are listed in Additional file 9: Table S9. Additional summarized GO annotations belonging to biological process are visualized in a TreeMap (REVIGO) (Additional file 10: Figure S1). “Peptidase activity” was an enriched GO term found in lice sampled from gills across all groups (except the ones exclusively found in Experiment 2 at 10 dpi), and in particular “serine type endopeptidase activity”, whereas “serine-type endopeptidase inhibitor activity” is enriched in lice sampled from skin. Notably, ”glycolysis” as well as “oxidoreductase activity” are GO terms highly enriched in transcripts elevated in lice sampled from gills. GO terms containing “phosphorylation” as well as “phosphatase” are found enriched in nearly all groups in genes elevated in lice sampled from skin (also here, the exceptions are the ones exclusively found in Experiment 2 at 10 dpi).

### Equal gene expression changes throughout all three analyses

**Fig. 5:**
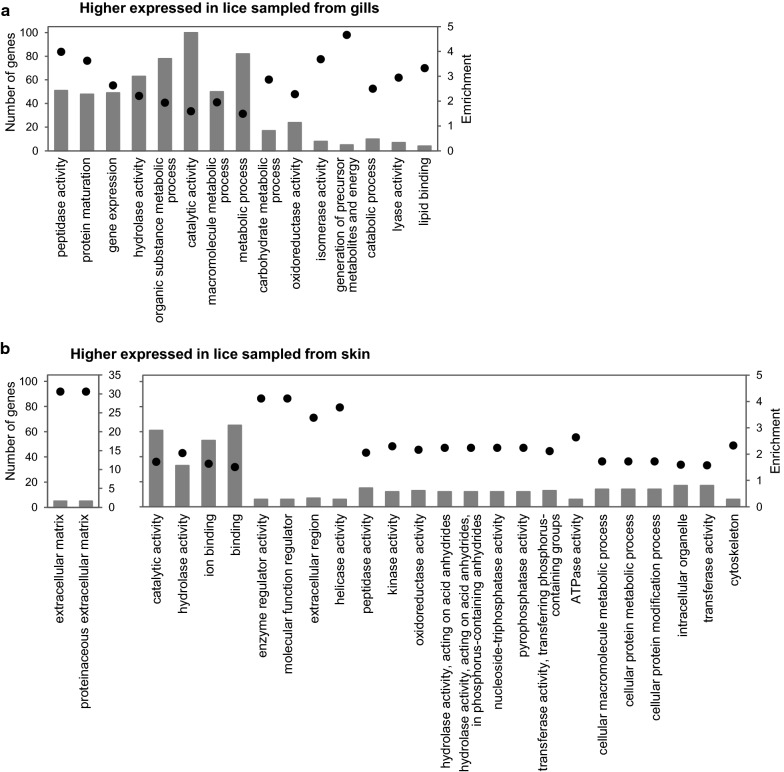
GOslim annotations of genes differently expressed in lice sampled from skin and gills. GO slim categories as well as enrichment of genes elevated in lice sampled from gills (**a**) and genes elevated in lice sampled from skin (**b**) in all three sampling points are shown (DESeq2; *P*adj < 0.05). Columns indicate the number of genes in each GOslim category and black dots show the enrichment of genes in that category in relation to number of genes of the specific category in the whole dataset

To determine which genes may be important in relation to the blood meal in general, independent of the stage of the lice at the different time points, we investigated the transcripts which were either significantly elevated in lice sampled from gills or significantly elevated in lice sampled from skin in all three samplings (Experiment 1 at 10 dpi, Experiment 2 at 10 dpi, Experiment 2 at 18 dpi) (DESeq2, *P*adj < 0.05). We found 355 transcripts that were elevated in all three samplings in lice from gills, and 202 from skin. Of the 355 genes elevated in lice sampled from gills, 60% had predicted Pfam domains, and of the 202 elevated in lice sampled from skin, 82% had predicted Pfam domains. A highly prevalent Pfam domain in the DE genes found in all 3 samplings elevated in lice sampled from gills is PF00089: Trypsin. Other frequently found domains found in lice from gills were PF01400: Astacin (Peptidase family M12A), PF02469: Fasciclin domain, PF05649: Peptidase family M13, PF00171: Aldehyde dehydrogenase family, as well as different Zinc finger domains. In the group of DE, genes which were elevated in lice sampled from skin, prevalent domains were PF00040: Fibronectin type II domain; PF00069: Protein kinase domain, PF00096: Zinc finger, PF00135: Carboxylesterase family, PF01391: Collagen triple helix repeat. A full list can be found in Additional file 4: Table S4 and Additional file 5: Table S5. GOslim was used to minimize GO categories. DE genes in lice sampled from gills fall under fewer GOslim categories than DE genes elevated in lice from skin, even though there were more genes in former DE group (Fig. 5). Genes in the group found elevated in lice sampled from gills frequently fell under the enriched GO group “catalytic activity” (all are shown in Fig. 5a). Remarkably strong enriched (factor 30) DE genes elevated in lice from skin, are genes belonging to “extracellular matrix”. However, only five genes were in this group. More than 30 genes were found in GO categories “catalytic activity”, “hydrolase activity”, “binding” and “ion binding” (Fig. 5b). All enriched GOslim terms, numbers of genes found in each category, and the enrichment factor for the two groups are shown in Fig. 5.

Forty-two percent of the 355 genes elevated in lice sampled from gills have a fold change of more than two compared to skin. When also taking the *P*-value into account we find 21%, which were strongly regulated (average fold change over two and average adjusted *P*-value ≤ 0.005). In this group, the strongest elevated transcript was most similar to a nematode astacin (EMLSAT00000010457). Among the other strong ones regulated were three transcripts containing FNII domains, another transcript with an astacin domain, as well as nine transcripts with trypsin domains, and a chemosensory protein (EMLSAT00000005105), together with many transcripts with no annotation or known protein domains. There were several transcripts with similarity to various proteases that were upregulated in lice from gills. All genes are listed in Additional file 4: Table S4. The expression patterns of the ten strongest regulated genes over all samples are shown in Additional file 11: Figure S2a.

In the group of genes elevated in lice sampled from skin, there were fewer genes highly differentially expressed than in lice sampled from gills. Only 16% have a fold change of two or more compared to samples from gills. Most upregulated in this group was a transcript with no predicted annotation or Pfam domains (EMLSAT00000009920). Among the strongest elevated genes in all three samplings (average fold change over two and *P*-value ≤ 0.005) in lice sampled from skin were several transcripts with predicted FNII domains. Moreover, there were several genes with no annotation or known protein domains. All genes are listed in Additional file 5: Table S5. Expression patterns for the ten strongest regulated genes are shown in Additional file 11: Figure S2b.

We further looked at the expression profile of these detected DE genes during the course of development, as well as in various tissues as published in [27, 59] or available from the salmon louse database LiceBase [60]. We were particularly interested in determining if these transcripts were also elevated in the louse intestine compared with other tissues, or if these transcripts are up- or downregulated after attachment or after molting to preadult, the expected time point for accessing host blood. Not all lice genes were represented on the microarray investigated by Edvardsen et al. [61]. Of the 202 genes elevated in lice from the skin, 124 were also present on the oligo microarray regarding expression in different tissues (gut adult female, gut adult male, ovaries, testis, subcuticular tissue and brain) and from the 355 genes elevated in lice sampled from gills 209 were represented in this microarray study [61]. Among transcripts elevated in lice sampled from gills, 94 (26%) were more highly expressed in the intestine and only 5% of transcripts were lowest in intestine compared with other tissues investigated (LiceBase [60]). Moreover, 39 of these were more than 100 times more highly expressed in the intestine, compared to other tissues (LiceBase [60]). Seventy-seven of these genes elevated in the intestine were also analyzed in the microarray study [61] and 52 of these genes were also found to be the most highly expressed genes in the intestine in that study. Sixty-six percent of the transcripts were elevated after attachment and 55% were more highly expressed in preadult lice than in chalimus II when comparing to time series data [27]. The genes more highly expressed in intestine, after attachment or in pad1 are marked in Additional file 4: Table S4.

Only 17 (8%) of the transcripts in the DE gene group that were elevated in lice sampled from skin were more highly expressed in the intestine than in other investigated tissues (12% lowest of all tissues investigated) (LiceBase [60]). Only two were more than 100 times more highly expressed in the intestine than other tissues (LiceBase [60]). However, the 10 of these which were also found on the oligo microarray were not most highly expressed in the intestine there, except for one (EMLSAT00000008355) [61]. In that study, two others were found highest expressed compared with the other tissues analyzed. Fifty-seven percent of the transcripts are elevated after attachment. Nearly all of the strongest regulated transcripts are elevated after attachment when comparing with LiceBase [60] data. Fifty-three percent are most highly expressed in preadult lice than chalimus II [27]. The genes most highly expressed in intestine, after attachment or in pad1, are marked in Additional file 5: Table S5.

## Discussion

In this study, we have investigated the biology of blood-feeding in the marine ectoparasitic salmon louse with a special focus on gene expression of immobile lice situated on host gills. We chose immobile lice, because this allowed us to focus on those individuals that had stayed at one location at least since extruding the frontal filament in the late copepodid stage. Being attached to the gills allowed the lice to initiate blood-feeding prior to becoming mobile. Samples from two experiments terminated at 10 days post-infestation, and one sample terminated at 18 days post-infestation of one the experiments were included in the RNA-seq and subsequent gene expression analyses.

### Distribution of lice

At 10 dpi, the number of lice was relatively evenly distributed between the investigated body parts (32 or 29% on the body of the host, 43 or 39% on the fins and 24 or 30% on gills in Experiment 1 and Experiment 2, respectively). The favored site at 18 dpi is the body with 66 or 61% in Experiment 1 or Experiment 2, respectively (15 or 14% on fins and 17 or 23% on gills) (Fig. 1, Table 2). Lice at day 10 were in chalimus I or chalimus II stage and attached by the frontal filament. On day 18 (Table 2), we found 68 and 66% preadult lice in Experiment 1 and Experiment 2, respectively, of which 10% (Experiment 1) and 21% (Experiment 2) were attached with a filament, while the others were mobile and could freely move on the fish. The finding that lice are differently distributed when mobile than attached, and mainly found on the body of the fish, suggests that the mobile preadult lice choose the general body surface as a preferred feeding site and migrate there from host fins and gills when becoming mobile. The majority (78% or 87%, in Experiment 1 and Experiment 2, respectively) of the preadult I lice on fins were females. Female lice are known to develop slower than males [4, 10], and this also indicates that the lice tend to leave the fins for other host feeding areas when becoming mobile.

### Onset of blood-feeding

A preadult I louse that is still attached to its host by its frontal filament has recently molted from the chalimus II stage and has stayed at that feeding site since attachment. There were no preadult I lice with a visible blood-filled intestine on the fins, whereas this was observed in lice on the gills and the body. Interestingly, of the lice still on their filament, only those on gills have apparently fed on blood. Moreover, already in the chalimus I stage, we found lice with blood-filled guts on the gills, but not at any other feeding site. As the preadult I lice on the body with a blood-filled intestine were mobile, these lice have either started with blood-feeding in the mobile preadult I stage or were preadult lice migrated from the gills, meaning that blood-feeding is initiated from the mobile preadult I stage and onwards in the development of the salmon louse occurring under field conditions. On gills however, even copepodids with blood-filled intestine can be found (personal observation, Additional file 12: Figure S3)) (not part of this study).

### Development of lice with regards to infestation site

Development of lice on the gills was delayed, compared to development of lice on body or fins. At 10 dpi, no chalimus II lice were found and a higher percentage of chalimus I lice was of less developed instar age on the gills. To compare the development of lice on the different body parts at 18 dpi, we were looking at the attached lice only, as these did not change place. Twenty-five percent of all lice collected at 18 dpi were attached chalimus II larvae. Fifty-one percent and 62% in Experiment 1 and Experiment 2, respectively, were found on gills. In addition, on gills, there was a higher proportion of male chalimus II lice, which develop faster than females. Lice on host gills showed reduced progress in development compared to lice settled at other locations. There have been contradictory results about this in the past [59, 62]; however, in this study we have determined instar ages, and not only the developmental stages, which adds more confidence to our results.

We conclude that during the normal development on the body or the fins, the salmon louse does not start to feed on blood until reaching the mobile preadult I stage. By that reasoning, we wanted to compare gene expression of chalimus larvae located on the vascular gills with access to blood with that of chalimus larvae equally developed from the rest of the body.

### Differences in the transcriptome

The salmon louse has approximately 13,000 protein-encoding genes (http://metazoa.ensembl.org/Lepeophtheirus_salmonis), and in our RNA-seq analyses over 5800 genes had an altered expression in at least one of our samplings. As expected, we found a high number of overlapping DE genes in the two samplings at 10 dpi. These were chalimus I larvae, which were soon molting to chalimus II, while lice sampled at 18 dpi were chalimus II larvae, shortly prior to molting to preadult I lice. As such, all lice were sampled at a similar instar age, namely short before molting (old). However, chalimus sampled at 18 dpi soon molt to preadult lice with a different phenotype and life style. One can expect expression of genes in preparation for the preadult stage in the lice sampled at 18 dpi which differ from the ones expressed in chalimus stage. The high number of DE genes exclusively found in Experiment 1 at 10 dpi could be caused by batch differences between Experiment 1 at 10 dpi and Experiment 2 at 10 dpi, or could be as a result of more powerful statistics due to a higher number of parallel samples (8 *versus* 6 biological parallels of each group in Experiment 1 at 10 dpi and Experiment 2 at 10 dpi, respectively). However, we know also that minor differences in development have a high impact on gene expression [27], and individual differences occurring within groups, with possible consequences between groups, could bias the results.

To investigate gene expression caused by nutritional differences, we mainly concentrated on the DE genes found in all three samplings. Transcripts overexpressed in lice sampled from gills could be important for hematophagy. However, many (70 of 74) of the strongest DE genes in this group were more highly expressed in tissues other than the intestine, suggesting that these genes contribute to other functions in the louse that may be modified by hematophagy. Genes elevated in lice from gills show a more homogenous GO annotation (fewer GOslim categories) than the ones elevated in lice from skin, suggesting that several DE genes are involved in the same processes. There were also more genes with a greater fold change within the group of DE genes elevated in lice sampled from gills (42% with over 2-fold change, whereas only 16% in lice sampled from skin), pointing towards a high demand for these gene products when feeding on blood. However, as GO terms can be unspecific or general, the following discussion deals with selected groups of transcripts.

### Iron and heme

Among the regulated transcripts, the iron storage units of ferritin (*LsFer1* (RACE sequence) and *LsFer2*: EMLSAT00000006305) were both elevated in chalimus larvae sampled from gills compared with other settlement sites (Additional file 13: Figure S4a, b). We have previously established that these genes are important for the adult female salmon louse blood-feeding and reproductive success, as the parasite had a clear gut and failed to produce viable eggs upon silencing these two genes [18]. Blood contains several iron-proteins, and when initiating blood-feeding, the salmon louse needs to obtain a way of storing and detoxifying iron absorbed from the blood. Upregulating ferritin when ingesting a blood meal is therefore an important defence mechanism for a blood-feeding parasite. The putative heme scavenger receptor, *LsHSCARB* (EMLSAT00000005382), is elevated in lice on gills at 18 dpi compared to lice on skin (Additional file 12: Figure S4d). We recently found that upon silencing *LsHSCARB* by RNA interference, adult female lice had absorbed less heme and produced fewer viable eggs and less offspring [17]. Lacking early (10 dpi) transcriptional elevation of *LsHSCARB* could indicate alternative mechanisms of absorption during the earlier developmental stages, or the existence of a post-transcriptional mode of regulating the LsHSCARB protein. Alternatively, the lack of early regulation might serve to maintain homeostasis of heme levels when feeding on the vascular gills.

### Detoxification

A glutathione S-transferase (GST) (PF02798) transcript (EMLSAT00000009830) was elevated in lice on gills in all samplings. GSTs are major detoxification enzymes. A GST in the hard tick *Ixodes ricinus* (IrGST1) (GenBank: MF984398) was also found to be elevated in the midgut of blood-fed ticks compared with serum-fed ticks [34]. Further characterization of IrGST1 showed that it was heme-inducible and the recombinant protein was able to bind heme *in vitro* [63]. The authors speculated that IrGST1 is important for detoxifying excess heme to avoid cytotoxicity in the tick [63]. Recombinant GSTX2 (GenBank: AAK64286.1) of *Ae. aegypti* also binds heme [64], and was elevated in a heme-incubated *Ae. aegypti* Aag2 cell line [29]. Of the six different predicted salmon louse proteins with the GST domain (PF02798), EMLSAP00000009830 is the most similar to both IrGST1 and *Ae. aegypti* GSTX2. The connection of GST and blood-feeding in the salmon louse is an interesting topic for future studies, as we currently do not know what mechanisms the salmon louse depends on to detoxify heme.

### Digestion

**Fig. 6:**
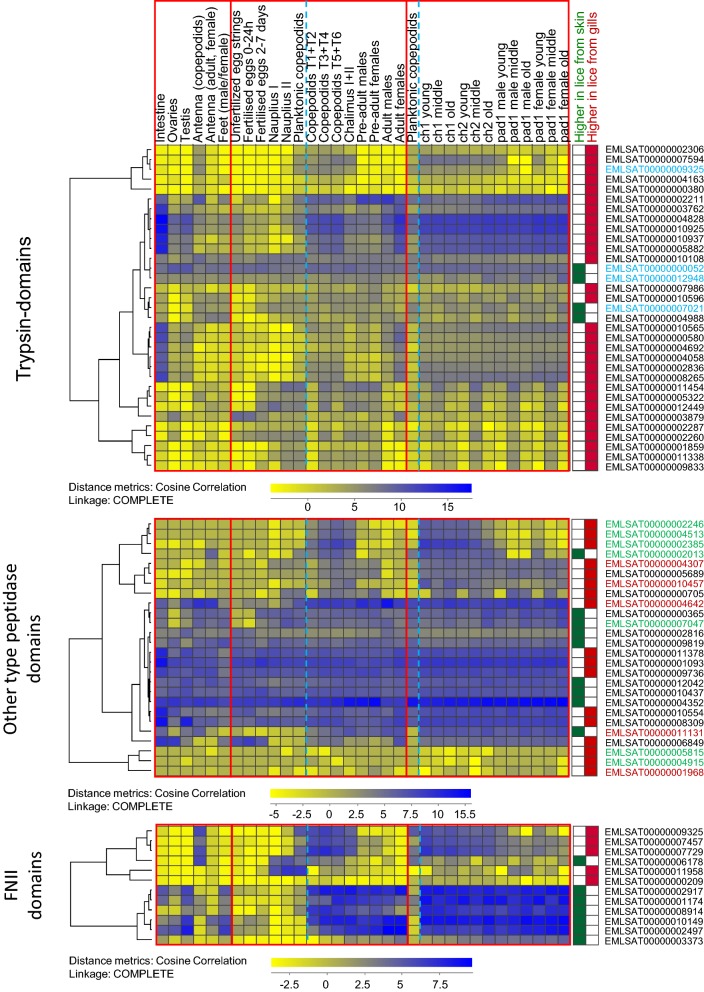
Expression of genes with special domains in different tissues and stages. The expression profiles of genes found differentially expressed in all three sampling points (DESeq2; *P*adj < 0.05) with trypsin, other peptidase and FNII domains in various tissues and stages (LiceBase [60] and from the time series study (average values of biological parallels) by Eichner et al. [27]) are shown in hierarchical clusters. A blue stippled line is separating planktonic and parasitic stages. *Key*: Stable IDs with font in blue, domains other than trypsin predicted as well; font in green, predicted M13 peptidases; font in red, predicted Astacin peptidases

Food protein hydrolysis is a fundamental step of digestion and is mediated by peptidases that enzymatically cleave peptide bonds. Blood is highly enriched in protein, and one of the most abundant ones is the gas transporter hemoglobin. Investigating changes in the salmon louse transcriptome upon initiating blood-feeding could thus give clues as to which enzymes are essential for the breakdown of blood components. Trypsin is a digestive enzyme belonging to the S1A subfamily of serine endopeptidases, and five main trypsin-encoding transcripts in the salmon louse intestine have previously been characterized [14, 15]. Trypsins and other proteins involved in protein degradation were found elevated, e.g. in blood-fed mosquito *Ae. aegypti* [28]. Twenty-eight transcripts with trypsin as the only predicted protein domain (PFAM: PF00089) (29 in total with trypsin + other domains) were found to be elevated in lice on host gills at day 10 (Experiment 1 and 2) and 18 dpi. Of these, 11 are predicted to be most highly expressed in the intestine compared with other tissues investigated in the salmon louse (LiceBase [60, 61]; Additional file 4: Table S4). A heat map showing the expression patterns for all transcripts with trypsin domains found DE in all three samplings in data taken from LiceBase [60] and from the time-series study [27] are shown in a hierarchical cluster in Fig. 6. *LsTryp1* (GenBank: AY294257, best BLAST hit: EMLSAT00000004828) was elevated in all three samplings in lice on gills. One transcript with a trypsin domain only (EMLSAT00000004988) was elevated in lice on host skin at both 10 dpi (Experiment 1 and Experiment 2) and at 18 dpi. However, RNA-seq data in LiceBase [60] as well as microarray data from Edvardsen et al. [57] show, that this transcript has a low expression in the louse intestine and is rather expressed in antenna and legs (annotated feet in LiceBase) [60] or subcuticular tissue and brain (microarray [61]). It might therefore be of importance for purposes other than blood-meal digestion.

Peptidases other than trypsins were also regulated in lice on host gills. There were 17 transcripts with Pfam domains “peptidase” other than trypsins elevated in lice on gills in all samplings. Among these are four transcripts with Astacin-domains (Peptidase family M12A) and five are M13 peptidases (Fig. 6). Both groups are metallopeptidases and are enriched in arthropods. Astacin-like metallopeptidases are implicated in digestive processes, but are also reported to have anticoagulative effects, as they are found to have fibrinogenolytic activity in spider venoms [65]. M13 metallopeptidases are widely distributed in animals, and e.g. make up the major group of the hematophagous tick degradome [66]. Furthermore, we also found many of the same types of peptidases elevated in lice from skin (one with Astacin domain, two with Peptidase family M13 domain). This could indicate different modes of digesting a blood meal *versus* digesting components of ingested salmon skin. Further investigation into the elevated trypsins and other peptidases expressed in the salmon louse gut should be conducted.

### Putative anti-coagulation

Blood coagulation is a key mechanism in maintaining homeostasis in vertebrates if a blood vessel were to rupture. A parasite feeding on vertebrate blood would therefore require mechanisms in order to counteract blood coagulation to maintain its feeding activity. Anti-coagulation factors targeting host proteins could thus be vital for the successful blood-feeding in the parasitizing arthropod. A thrombin (coagulation factor) inhibitor, hemalin, was found to be important to avoid clotting of the blood meal in the bush tick *Haemaphysalis longicornis* [67]. A salmon louse transcript (EMLSAT00000003009) encoding two Kunitz/Bovine pancreatic trypsin inhibitor domains (PF00014), as also found in the tick hemalin, was elevated here in all three samplings in lice on gills. However, four other transcripts with the same domain were elevated in lice from skin in all samplings (EMLSAT00000000152, EMLSAT00000007907, EMLSAT00000008877 and EMLSAT00000009255).

We also find serine protease inhibitors (serpins, PF00079) regulated. From the 15 predicted serpin transcripts in the louse, four were DE in all three samplings. Two were elevated in lice on gills (EMLSAT00000010931 and EMLSAT00000001743), one elevated in lice on skin (EMLSAT00000011353), while the last (EMLSAT00000005224) was expressed lower in lice sampled from gills at 10 dpi, but elevated at 18 dpi. One transcript (EMLSAT00000000552) was elevated in lice on gills at 10 dpi only (Experiment 1 and Experiment 2). Anti-coagulation factors could be targets for pest control as they are likely secreted and in contact with the host, and thus probably vital for the host-parasite interaction.

### Fibronectin type II

The approximately 60 amino acid long fibronectin type II (FNII) domain (PF00040) is a protein domain found within the glycoprotein fibronectin. It contains four conserved cysteine residues that form disulfide bridges. These residues are important for e.g. fibronectin’s collagen binding properties [68]. The FNII domain is also found within the vertebrate blood coagulation protein Factor XII [69]. The FNII domain is the most expanded protein domain of the salmon louse with over 200 copies within over 80 genes identified so far [40] (*Lepeophtheirus salmonis*, LSalAtl2s). Some of these genes (*LsFNII1*, *2* and *3*) have been characterized, and are expressed in tegumental type 1 (teg1) glands of the salmon louse [70, 71]. Teg1 glands are exocrine and their secretory ducts are extending to the dorsal and ventral side of the salmon louse [71]. The functions of FNII-containing proteins have not been determined in the salmon louse; however, it has been suggested that proteins with the domain may be of importance for lubricating the integument and may function asanti-fouling agent, or as part of the salmon louse fuzzy coat (acid mucopolysaccharide layer [72]) [70]. Genes with FNII domains expressed in teg1 glands have also been suggested to be of importance for host immune modulation by the parasite [71].

Several FNII-containing genes were significantly (DESeq2, *P*adj < 0.05) regulated in chalimus larvae in this study. Five transcripts with predicted FNII domains were elevated in all three samplings in lice on gills. Of these, all but one (EMLSAT00000011958) are predicted to be upregulated after louse attachment (LiceBase [60]; Additional file 4: Table S4) (Fig. 6). Seven transcripts with FNII were found elevated in lice on skin in all three experiments. Here as well, all but one transcript (EMLSAT00000006178) are predicted to be upregulated after louse attachment (LiceBase [60]; Additional file 5: Table S5). The FNII encoding genes characterized by Øvergård et al. [71] and Harasimczuk et al. [70] (*LsFNII1*: EMLSAT00000012082, *LsFNII2*: EMLSAT00000007294, *LsFNII3*: EMLSAT00000009744) are not among the transcripts regulated in all samplings here. However, LsFNII1 was elevated in both experiments at 10 dpi, and LsFNII3 was elevated at 18 dpi as well as in Exp1 10 dpi in lice sampled from gills. The transcripts elevated in lice on gills should be further characterized, in order to elucidate a possible role of FNII in blood-feeding. Given the earlier reports that FNII domains in vertebrates may be important for blood clotting, one hypothesis is that proteins containing FNII domains only could have an anti-coagulant effect.

### Blood-feeding *versus* infection site

We found large differences between lice from different settlement sites and we assume that a large part of the variation can be explained by differences in food composition such as nutrients, pathogens and exposure to host defenses. However, it has to be taken into account that settlement sites could have other influential factors such as differences in water flow or microbiome. Within in the confines of this study, we are unable to differentiate between these possible factors. More studies will be necessary to elucidate potential confounding effects of the gill environment. It also has to be taken into account, that gill settlement is rare in the field. It cannot be excluded, that early larvae are less adapted to a hematophagous life style. On the one hand, this might cause elevated levels of stress response (false positives), on the other hand, developmental regulatory mechanism might prohibit an otherwise regulatory response to the blood meal (false negatives). It is for example possible that some genes like the scavenger receptor LsHSCARB or other genes which were upregulated in lice on gills only at 18 dpi but not at 10 dpi are inhibited by an additional developmental regulator until a certain stage and their positive co-regulation with blood presence can only be activated after the developmental stage is reached.

## Conclusions

Blood is a major dietary component for the ectoparasitic salmon louse, which the parasite has access to when attached to a salmonid host. We found that the salmon louse initiates blood-feeding during the mobile preadult I stage. However, if the parasite is attached to host gills, it may start feeding on blood already at the chalimus I stage or even earlier. Blood can be found even in copepodids sampled from gills. The premature onset of blood-feeding caused lice on gills to develop at a slower pace than lice that were attached to host fins and general body surfaces. Chalimus lice of equivalent age on gills *versus* other attachment sites were therefore analyzed for gene expression comparisons. Several genes were elevated in lice attached to the gills, and among these, we found, e.g. genes of importance for the absorption, storage and/or transportation of the pro-oxidative molecules iron and heme, digestive and detoxification enzymes, genes that could be important for anti-clotting of host blood and several genes with FNII domains. The results of this study highlight a number of new gene targets to investigate further in order to elucidate the blood-feeding habits of the infamous salmon louse.

## Supplementary information

**Additional file 1: Table S1.** Results from all DESeq2 analyses for all genes analyzed.

**Additional file 2: Table S2.** CPM values of all transcripts included in this study for the three different samplings: Experiment 1 (10 dpi), Experiment 2 (10 dpi) and Experiment 2 (18 dpi) (38 samples).

**Additional file 3: Table S3.** Genes, at least in one of the samplings differentially expressed (DESeq2, *P*adj < 0.05).

**Additional file 4: Table S4.** Genes differentially expressed in all 3 samplings (DESeq2, *P*adj < 0.05). Higher expressed in lice sampled from gills.

**Additional file 5: Table S5.** Genes differentially expressed in all 3 samplings (DESeq2, *P*adj < 0.05). Higher expressed in lice sampled from skin.

**Additional file 6: Table S6.** Genes differentially expressed in all 3 samplings (DESeq2, *P*adj < 0.05) but with different sign in different samplings.

**Additional file 7: Table S7.** Genes differentially expressed (DESeq2, *P*adj < 0.05) in both samplings taken at 10 dpi but not at 18 dpi.

**Additional file 8: Table S8.** Genes differentially expressed (DESeq2, *P*adj < 0.05) at 18 dpi only.

**Additional file 9: Table S9.** Enriched GO terms for genes shown in the different compartments of the Venn diagrams (Fig. 4).

**Additional file 10: Figure S1.** Tree maps (Revigo) of enriched GO annotation belonging to biological process.

**Additional file 11: Figure S2.** Expression profiles of the strongest regulated genes (DESeq2; *P*adj < 0.005, average fold-change > 2).

**Additional file 12: Figure S3.** Copepodite with blood *versus* not blood-filled intestine.

**Additional file 13: Figure S4.** Expression of ferritins, LsHSCARB and a lipid transporter gene.

## Data Availability

The datasets supporting the conclusions of this article are included within the article and its additional files. Raw RNA-sequencing data files have been deposited to NCBI BioProject under the accession number PRJNA577842.

## Abbreviations

RNA-seq: RNA-sequencing
dpi: day(s) post-infestation
TL: total length
CL: cephalothorax length
Exp: experiment
RACE: rapid amplification of cDNA ends
DE: differential expression
GO: Gene Ontology
pad: preadult
ch: chalimus
CPM: counts per million
CA: correspondence analysis
PCA: principal components analysis
FDR: false discovery rate
*P*adj: adjusted *P*-value
teg1: tegumental type 1
