# Supplementary material for: Host gill attachment causes blood-feeding by the salmon louse (Lepeophtheirus salmonis) chalimus larvae and alters parasite development and transcriptome
Source: Parasit Vectors. 2020 May 6;13:225. doi: 10.1186/s13071-020-04096-0 (PMC7201535; doi:10.1186/s13071-020-04096-0)
Supplement: Supplementary file 10 — Additional file 10: Figure S1. Tree maps (Revigo) of enriched GO annotation belonging to biological process. [file 13071_2020_4096_MOESM10_ESM.pdf]

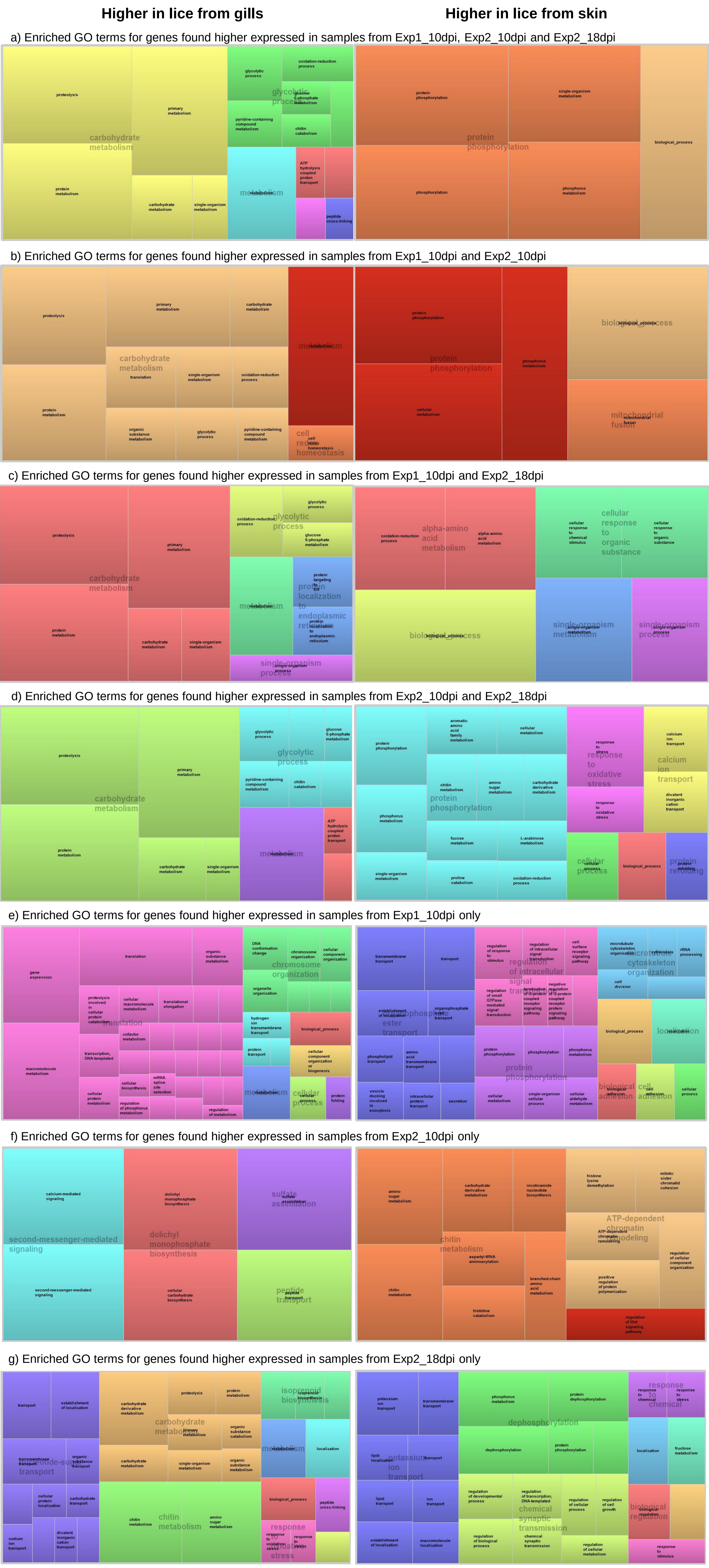

Figure S1. Tree maps (Revigo) of enriched GO annotation belonging to biological process. Shown are tree maps for GO annotations enriched in genes found higher expressed in samples from all three samplings (DESeq2; padj < 0.05) (a), of the genes differentially expressed in both samplings at 10 days post-infestation (dpi) (b); and in 2 samplings sampled at different time point (c and d). In e- g are enriched GO annotations shown for genes differentially expressed in one of the three samplings only.
