# Supplementary material for: Host gill attachment causes blood-feeding by the salmon louse (Lepeophtheirus salmonis) chalimus larvae and alters parasite development and transcriptome
Source: Parasit Vectors. 2020 May 6;13:225. doi: 10.1186/s13071-020-04096-0 (PMC7201535; doi:10.1186/s13071-020-04096-0)
Supplement: Supplementary file 11 — Additional file 11: Figure S2. Expression profiles of the strongest regulated genes (DESeq2; Padj < 0.005, average fold-change > 2). [file 13071_2020_4096_MOESM11_ESM.pdf]

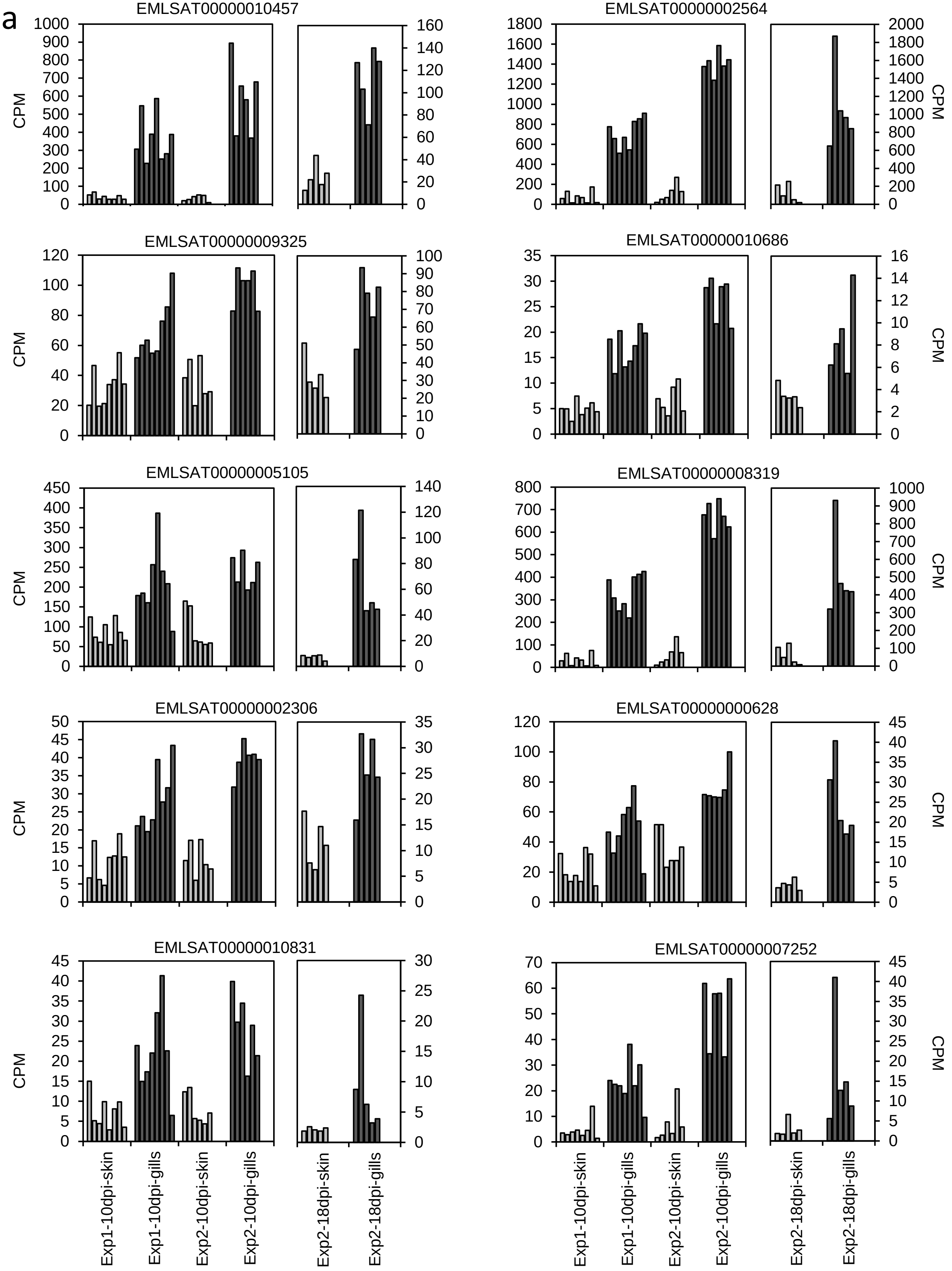
a
EMLSAT00000010457
EMLSAT00000002564
EMLSAT00000009325
EMLSAT00000010686
EMLSAT00000005105
EMLSAT00000008319
EMLSAT00000002306
EMLSAT00000000628
EMLSAT00000010831
EMLSAT00000007252
CPM
Exp1-10dpi-skin
Exp1-10dpi-gills
Exp2-10dpi-skin
Exp2-10dpi-gills
Exp2-18dpi-skin
Exp2-18dpi-gills

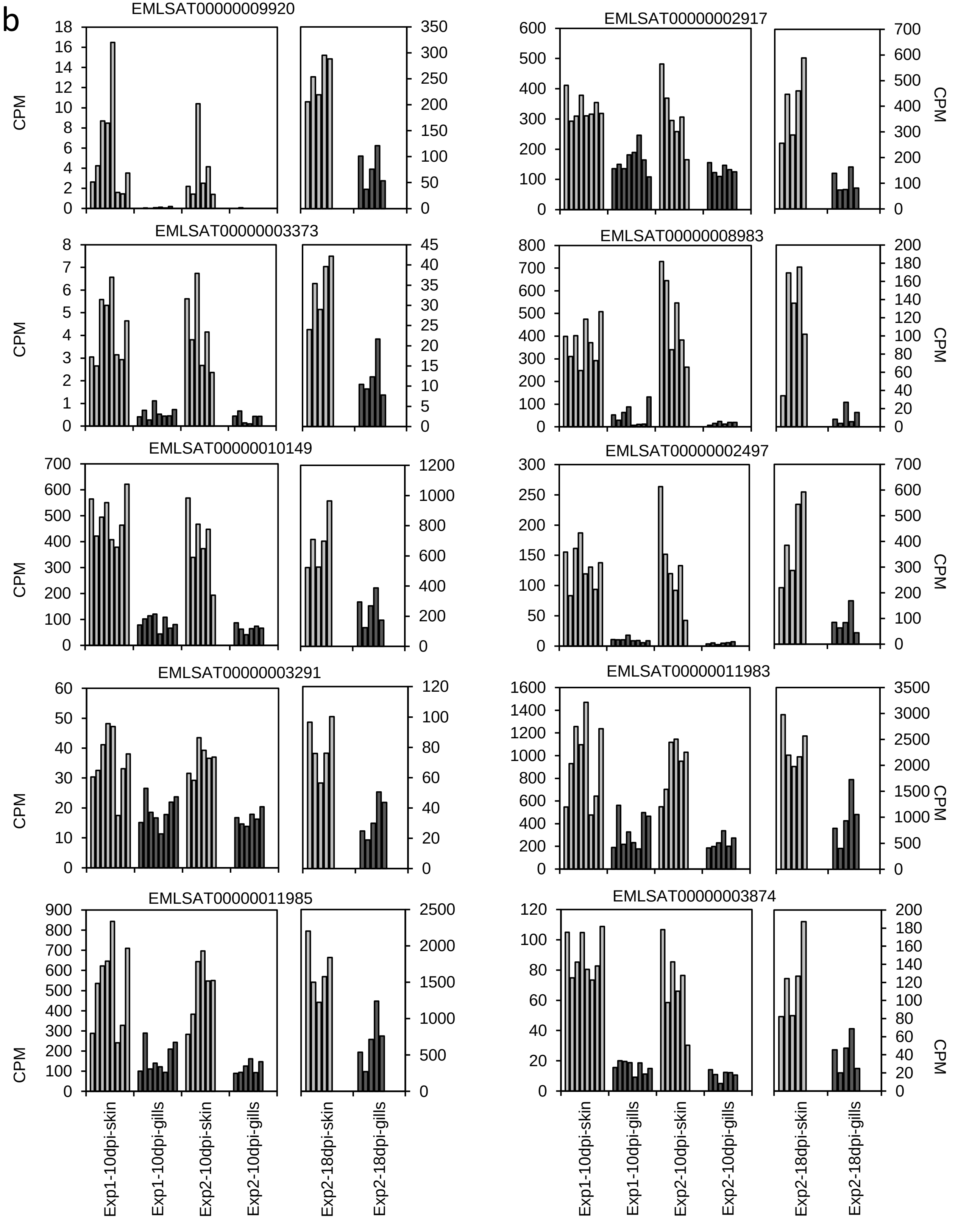

Figure S2. Expression profiles of strongest regulated genes. The expression profiles over all samples of the ten strongest genes (DESeq2; padj < 0.005, average fold-change > 2) upregulated in lice sampled from gills (a) and in lice sampled from skin (b).
