# Supplementary material for: Host gill attachment causes blood-feeding by the salmon louse (Lepeophtheirus salmonis) chalimus larvae and alters parasite development and transcriptome
Source: Parasit Vectors. 2020 May 6;13:225. doi: 10.1186/s13071-020-04096-0 (PMC7201535; doi:10.1186/s13071-020-04096-0)
Supplement: Supplementary file 12 — Additional file 12: Figure S3. Copepodite with blood versus not blood-filled intestine. [file 13071_2020_4096_MOESM12_ESM.pdf]

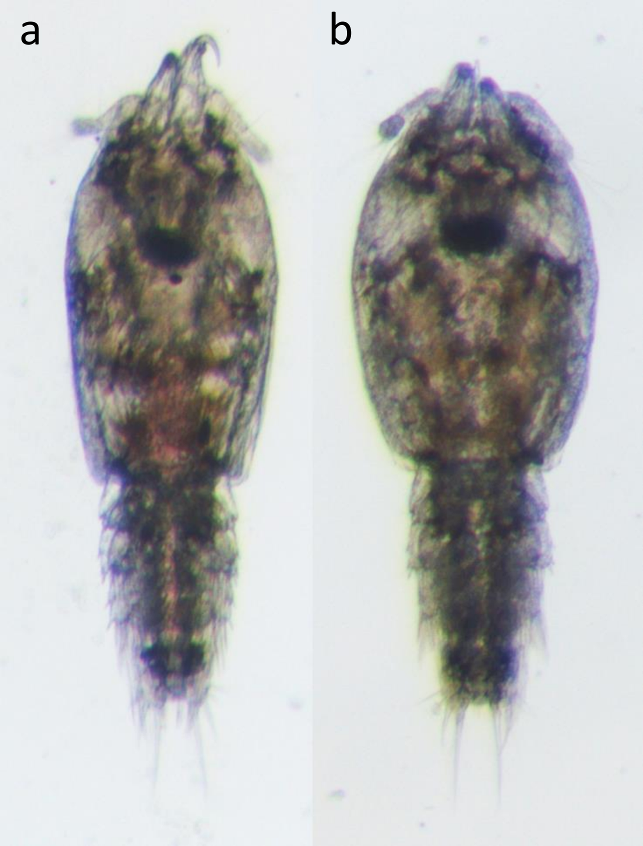

Figure S3. Copepodite with blood *versus* not blood filled intestine. The photographs show copepodites sampled at three days post-infestation with blood (a), sampled from gills and without blood (b) (samples not included in this study).
