# Supplementary material for: Host gill attachment causes blood-feeding by the salmon louse (Lepeophtheirus salmonis) chalimus larvae and alters parasite development and transcriptome
Source: Parasit Vectors. 2020 May 6;13:225. doi: 10.1186/s13071-020-04096-0 (PMC7201535; doi:10.1186/s13071-020-04096-0)
Supplement: Supplementary file 13 — Additional file 13: Figure S4. Expression of ferritins, LsHSCARB and a lipid transporter gene. [file 13071_2020_4096_MOESM13_ESM.pdf]

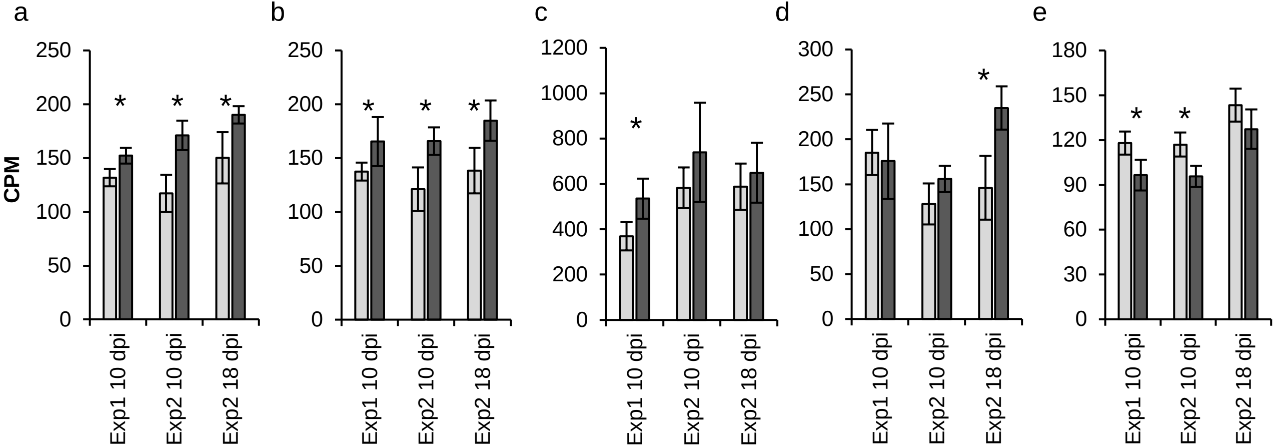

Figure S4. Expression of ferritins, LsHSCARB and a lipid transporter gene. a) Ferritin 1, b) Ferritin 2, c) Ferritin 4, d) LsHSCARB, e) Lipid transfer protein (EMLSAT00000001530) (Khan et al., 2017) in the samples from lice sampled from skin (light grey) and gills (dark grey). Shown are average values with standard deviation. * = Significant different (T-test, $p < 0.05$) between lice samples from different location in the respective sampling point.
